# *N*-[2-(2-Hy­droxy­eth­oxy)pheneth­yl]phthalimide

**DOI:** 10.1107/S1600536812018429

**Published:** 2012-05-05

**Authors:** Er-Qun Yang, Jun-Tao Zhang, Xiao-Ping Cao, Jin-Zhong Gu

**Affiliations:** a State Key Laboratory of Applied Organic Chemistry and College of Chemistry and Chemical Engineering, Lanzhou University, Lanzhou, Gansu 730000, People’s Republic of China; b Key Laboratory of Nonferrous Metal Chemistry and Resources, Utilization of Gansu Province, College of Chemistry and Chemical Engineering, Lanzhou University, Lanzhou, Gansu 730000, People’s Republic of China

## Abstract

The title compound, C_18_H_17_NO_4_, was obtained accidentally through acid-catalysed aromatization of a phthalimide-substituted 2-(1-hy­droxy­eth­yl)cyclo­hex-2-enone. It exhibits an intra­molecular O—H⋯O_c_ (c = carbonyl) hydrogen bond and forms a three-dimensional network structure *via* π–π stacking inter­actions between adjacent benzene rings (phthalimide-to-phenyl­ene and phthalimide-to-phthalimide), with centroid–centroid distances of 3.8262 (6) and 3.6245 (5) Å.

## Related literature

For background to the titanium(IV) chloride-promoted Baylis–Hillman reaction, see: Basavaiah *et al.* (2010 ▶); Park *et al.* (2004 ▶); Qi *et al.* (2011 ▶); Reggelin *et al.* (2006 ▶); Veale *et al.* (2008 ▶). For protection of ketones as 1,3-dioxolanes, see: Chen *et al.* (2011 ▶); Shih & Swenton (1982 ▶). For background and a possible mechanism of the aromatization reaction, see: Patra *et al.* (2002 ▶); Lewin *et al.* (2008 ▶).
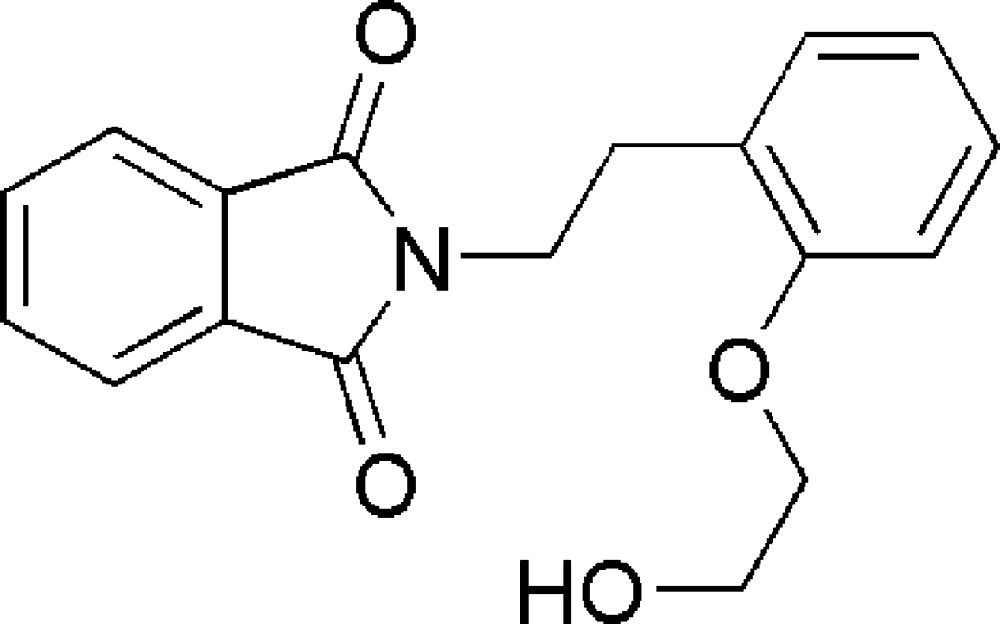


## Experimental

### 

#### Crystal data

C_18_H_17_NO_4_
*M* *_r_* = 311.33
Monoclinic, 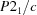

*a* = 8.4799 (19) Å
*b* = 22.954 (5) Å
*c* = 8.5089 (19) Å
β = 110.077 (2)°
*V* = 1555.6 (6) Å^3^
*Z* = 4
Mo *K*α radiation
μ = 0.09 mm^−1^
*T* = 296 K
0.32 × 0.29 × 0.21 mm

#### Data collection

Bruker APEXII CCD diffractometer
Absorption correction: multi-scan (*SADABS*; Bruker, 2004 ▶) *T*_min_ = 0.970, *T*_max_ = 0.980
10978 measured reflections
2891 independent reflections
1867 reflections with *I* > 2σ(*I*)
*R*_int_ = 0.039

#### Refinement

*R*[*F* ^2^ > 2σ(*F* ^2^)] = 0.054
*wR*(*F* ^2^) = 0.146
*S* = 1.04
2891 reflections
209 parameters
H-atom parameters constrained
Δρ_max_ = 0.27 e Å^−3^
Δρ_min_ = −0.25 e Å^−3^

### 

Data collection: *APEX2* (Bruker, 2004 ▶); cell refinement: *SAINT* (Bruker, 2004 ▶); data reduction: *SAINT*; program(s) used to solve structure: *SHELXS97* (Sheldrick, 2008 ▶); program(s) used to refine structure: *SHELXL97* (Sheldrick, 2008 ▶); molecular graphics: *SHELXTL* (Sheldrick, 2008 ▶); software used to prepare material for publication: *SHELXTL*.

## Supplementary Material

Crystal structure: contains datablock(s) I, global. DOI: 10.1107/S1600536812018429/zl2473sup1.cif

Structure factors: contains datablock(s) I. DOI: 10.1107/S1600536812018429/zl2473Isup2.hkl

Supplementary material file. DOI: 10.1107/S1600536812018429/zl2473Isup3.cml

Additional supplementary materials:  crystallographic information; 3D view; checkCIF report

## Figures and Tables

**Table 1:** Hydrogen-bond geometry (Å, °)

*D*—H⋯*A*	*D*—H	H⋯*A*	*D*⋯*A*	*D*—H⋯*A*
O4—H4*A*⋯O2	0.82	2.14	2.941 (3)	164

**Fig. 1.**
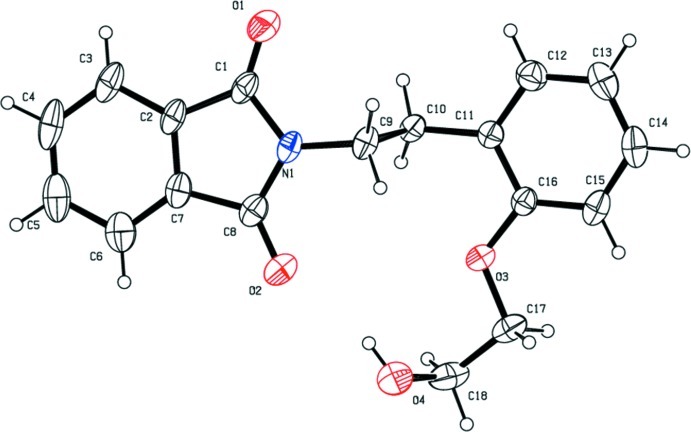
A view of the title compound, showing the atom-numbering scheme. Displacement ellipsoids drawn at the 50% probability level.

**Fig. 2.**
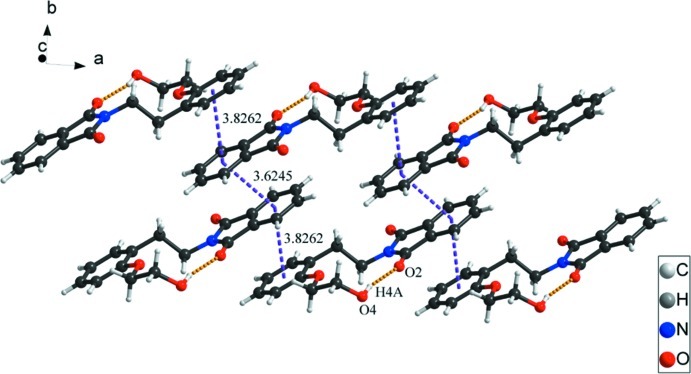
Molecular packing of the title compound, viewed down the c axis, showing intramolecular O—H···O hydrogen bonds as yellow lines and π-π interactions as purple lines.

**Fig. 3.**
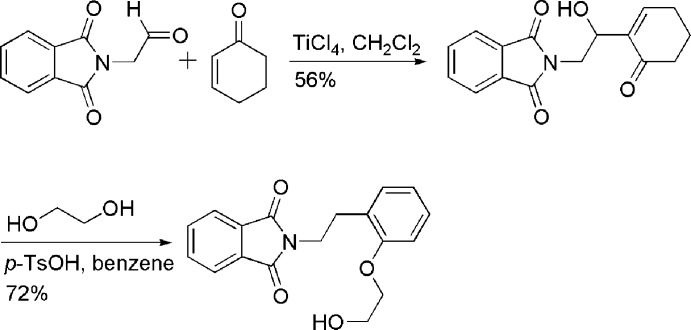
Synthesis of the title compound.

## supplementary crystallographic information

### Comment

*N*-[2-(2-Hydroxyethoxy)phenethyl]phthalimide (Fig. 1), was accidentally obtained as an unintended product from a total synthesis of malyngamides. In the first step *N*-[(formyl)methyl]phthalimide was reacted with cyclohex-2-enone *via* a titanium (IV) chloride promoted Baylis-Hillman reaction. The 2-(1-hydroxyethyl)cyclohex-2-enone obtained was then reacted with ethylene glycol with *p-*toluenesulfonic acid as the catalyst, with the aim to protect the keto group as a 1,3-dioxolane (Chen *et al.*, 2011; Shih & Swenton, 1982). However, condensation with ethylene glycol proofed incomplete and through elimination of the hydroxy group and subsequent aromatization of the cyclohex-2-enone ring through a [1,5] shift of the double bond the title compound was obtained instead (Fig. 3). A similar reaction involving an aromatization of a cyclohex-2-enone obtained *via* a titanium (IV) chloride promoted Baylis-Hillman Reaction had been described earlier by *e.g.* Patra *et al.* (2002). This unexpected reaction offers itself as a good strategy for the synthesis of 2-substituted β-phenethylamines which are amino acid metabolites and important intermediates in medicinal chemistry (Lewin *et al.*, 2008).

As shown in Fig. 2, an intramolecular O—H···O hydrogen bond is formed between the hydroxy group and one of the keto oxygen atoms (Table 1). In the crystal, the crystal packing is further stabilized by π-π interactions between phenyl rings in neighboring molecules (phthalimide to phenylene and phthalimide to phthalimide), with centroid to centroid distances of 3.8262 (6) Å and 3.6245 (5) Å.

### Experimental

The title compound was produced in two steps. Using Baylis-Hillman reaction conditions (Basavaiah *et al.*, 2010; Park *et al.*, 2004), *N*-[2-hydroxy-2-(6-oxocyclohex-1-enyl)ethyl]phthalimide was prepared from *N*-[(formyl)methyl]phthalimide (Qi *et al.*, 2011; Reggelin *et al.*, 2006; Veale *et al.*, 2008) and cyclohex-2-enone with titanium (IV) chloride in 56% yield. Then, to a stirred solution of *N*-[2-hydroxy-2-(6-oxocyclohex-1-enyl)ethyl]phthalimide (163 mg) in benzene (10 ml), ethylene glycol (4.40 ml) and *p*-toluenesulfonic acid (1 mg) were added and the mixture was refluxed for 20 h. The reaction mixture was poured into saturated NaHCO_3_ solution (10 ml), extracted with Et_2_O (3 × 20 ml) and then dried over MgSO_4_. The solvent was removed under reduced pressure, and the residue was purified by flash column chromatography on silica gel to give the title compound (128 mg, 72%) as a colourless solid. m.p. 403–404 K; ^1^H NMR (CDCl_3_, 400 MHz) δ: 7.85 (m, 2H, ArH), 7.71 (m, 2H, ArH), 7.22 (t, J = 7.4 Hz, 2H, ArH), 6.88 (m, J = 7.4 and 8.8 Hz, 2H, ArH), 4.07 (m, 4H, CH_2_), 3.92 (m, 2H, CH_2_), 2.98 (t, J = 8.4 Hz, 2H, CH_2_); ^13^C NMR (CDCl_3_, 100 MHz) δ: 168.6 (C), 157.0 (C), 134.0 (CH, overlapping signals), 132.1 (C), 130.9 (CH), 128.3 (CH), 126.0 (C), 123.4 (CH, overlapping signals), 120.8 (CH), 111.0 (CH), 69.5 (CH_2_), 61.5 (CH_2_), 37.9 (CH_2_), 30.6 (CH_2_); MS (ESI) m/*z* (%): 311 (*M*+, 30), 281 (9), 164 (100), 160 (53), 133 (73), 120 (57).

### Refinement

All H atoms were placed in geometrically idealized positions, with C—H = 0.93 Å and O—H = 0.82 Å, and constrained to ride on their respective parent atoms, with *U*_iso_(H) = 1.2Ueq(C) and *U*_iso_(H) = 1.5Ueq(O).

### Crystal data

**Table:**

C_18_H_17_NO_4_	*F*(000) = 656
*M**_r_* = 311.33	*D*_x_ = 1.329 Mg m^−^^3^
Monoclinic, *P*2_1_/*c*	Mo *K*α radiation, λ = 0.71073 Å
*a* = 8.4799 (19) Å	Cell parameters from 3059 reflections
*b* = 22.954 (5) Å	θ = 2.6–27.7°
*c* = 8.5089 (19) Å	µ = 0.09 mm^−^^1^
β = 110.077 (2)°	*T* = 296 K
*V* = 1555.6 (6) Å^3^	Block, colourless
*Z* = 4	0.32 × 0.29 × 0.21 mm

### Data collection

**Table:**

Bruker APEXII CCD diffractometer	2891 independent reflections
Radiation source: fine-focus sealed tube	1867 reflections with *I* > 2σ(*I*)
Graphite monochromator	*R*_int_ = 0.039
φ and ω scans	θ_max_ = 25.5°, θ_min_ = 2.6°
Absorption correction: multi-scan (*SADABS*; Bruker, 2004)	*h* = −10→10
*T*_min_ = 0.970, *T*_max_ = 0.980	*k* = −27→26
10978 measured reflections	*l* = −9→10

### Refinement

**Table:**

Refinement on *F*^2^	Primary atom site location: structure-invariant direct methods
Least-squares matrix: full	Secondary atom site location: difference Fourier map
*R*[*F*^2^ > 2σ(*F*^2^)] = 0.054	Hydrogen site location: inferred from neighbouring sites
*wR*(*F*^2^) = 0.146	H-atom parameters constrained
*S* = 1.04	*w* = 1/[σ^2^(*F*_o_^2^) + (0.0492*P*)^2^ + 0.9244*P*] where *P* = (*F*_o_^2^ + 2*F*_c_^2^)/3
2891 reflections	(Δ/σ)_max_ < 0.001
209 parameters	Δρ_max_ = 0.27 e Å^−^^3^
0 restraints	Δρ_min_ = −0.25 e Å^−^^3^

### Special details

**Table:**

Geometry. All e.s.d.'s (except the e.s.d. in the dihedral angle between two l.s. planes) are estimated using the full covariance matrix. The cell e.s.d.'s are taken into account individually in the estimation of e.s.d.'s in distances, angles and torsion angles; correlations between e.s.d.'s in cell parameters are only used when they are defined by crystal symmetry. An approximate (isotropic) treatment of cell e.s.d.'s is used for estimating e.s.d.'s involving l.s. planes.
Refinement. Refinement of *F*^2^ against ALL reflections. The weighted *R*-factor *wR* and goodness of fit *S* are based on *F*^2^, conventional *R*-factors *R* are based on *F*, with *F* set to zero for negative *F*^2^. The threshold expression of *F*^2^ > σ(*F*^2^) is used only for calculating *R*-factors(gt) *etc*. and is not relevant to the choice of reflections for refinement. *R*-factors based on *F*^2^ are statistically about twice as large as those based on *F*, and *R*- factors based on ALL data will be even larger.

### Fractional atomic coordinates and isotropic or equivalent isotropic displacement parameters (Å^2^)

**Table:**

	*x*	*y*	*z*	*U*_iso_*/*U*_eq_	
C1	0.3135 (3)	0.43925 (12)	0.1572 (4)	0.0526 (7)	
C2	0.4828 (3)	0.45631 (11)	0.2714 (4)	0.0510 (7)	
C3	0.5941 (4)	0.49717 (13)	0.2529 (4)	0.0678 (9)	
H3	0.5698	0.5191	0.1556	0.081*	
C4	0.7434 (4)	0.50426 (16)	0.3846 (6)	0.0807 (11)	
H4	0.8209	0.5315	0.3757	0.097*	
C5	0.7792 (4)	0.47205 (16)	0.5276 (5)	0.0784 (11)	
H5	0.8809	0.4778	0.6139	0.094*	
C6	0.6675 (4)	0.43115 (14)	0.5464 (4)	0.0656 (8)	
H6	0.6920	0.4091	0.6436	0.079*	
C7	0.5189 (3)	0.42441 (11)	0.4159 (4)	0.0502 (7)	
C8	0.3739 (3)	0.38582 (11)	0.3991 (4)	0.0500 (7)	
C9	0.0948 (3)	0.36841 (11)	0.1771 (3)	0.0502 (7)	
H9A	0.1011	0.3308	0.2308	0.060*	
H9B	0.0678	0.3617	0.0581	0.060*	
C10	−0.0443 (3)	0.40388 (10)	0.2042 (3)	0.0435 (6)	
H10A	−0.0109	0.4153	0.3209	0.052*	
H10B	−0.0623	0.4391	0.1373	0.052*	
C11	−0.2049 (3)	0.36999 (10)	0.1575 (3)	0.0404 (6)	
C12	−0.3276 (3)	0.37546 (13)	0.0032 (3)	0.0581 (7)	
H12	−0.3116	0.4017	−0.0731	0.070*	
C13	−0.4738 (4)	0.34333 (15)	−0.0424 (4)	0.0710 (9)	
H13	−0.5541	0.3475	−0.1484	0.085*	
C14	−0.4990 (4)	0.30551 (14)	0.0690 (4)	0.0681 (9)	
H14	−0.5974	0.2837	0.0389	0.082*	
C15	−0.3807 (3)	0.29891 (12)	0.2265 (4)	0.0575 (7)	
H15	−0.3995	0.2733	0.3029	0.069*	
C16	−0.2334 (3)	0.33096 (10)	0.2697 (3)	0.0425 (6)	
C17	−0.1242 (4)	0.29088 (13)	0.5486 (4)	0.0621 (8)	
H17A	−0.1393	0.2507	0.5108	0.075*	
H17B	−0.2202	0.3025	0.5783	0.075*	
C18	0.0337 (5)	0.29740 (15)	0.6943 (4)	0.0761 (10)	
H18A	0.0512	0.3383	0.7242	0.091*	
H18B	0.0229	0.2766	0.7892	0.091*	
N1	0.2576 (2)	0.39694 (9)	0.2431 (3)	0.0467 (5)	
O1	0.2337 (3)	0.45710 (10)	0.0197 (3)	0.0780 (7)	
O2	0.3549 (3)	0.35172 (9)	0.4993 (3)	0.0696 (6)	
O3	−0.1065 (2)	0.32785 (8)	0.4213 (2)	0.0543 (5)	
O4	0.1750 (3)	0.27578 (10)	0.6595 (3)	0.0903 (8)	
H4A	0.2069	0.3003	0.6069	0.136*	

### Atomic displacement parameters (Å^2^)

**Table:**

	*U*^11^	*U*^22^	*U*^33^	*U*^12^	*U*^13^	*U*^23^
C1	0.0511 (16)	0.0515 (17)	0.072 (2)	0.0004 (13)	0.0424 (15)	0.0039 (15)
C2	0.0478 (15)	0.0466 (15)	0.0762 (19)	−0.0039 (12)	0.0438 (14)	−0.0084 (14)
C3	0.063 (2)	0.0561 (18)	0.109 (3)	−0.0071 (15)	0.061 (2)	−0.0051 (18)
C4	0.059 (2)	0.071 (2)	0.138 (3)	−0.0224 (17)	0.067 (2)	−0.040 (2)
C5	0.0486 (19)	0.085 (3)	0.113 (3)	−0.0090 (17)	0.041 (2)	−0.044 (2)
C6	0.0549 (18)	0.070 (2)	0.079 (2)	0.0012 (16)	0.0325 (17)	−0.0209 (17)
C7	0.0442 (15)	0.0469 (15)	0.0706 (19)	−0.0007 (12)	0.0342 (14)	−0.0151 (14)
C8	0.0539 (16)	0.0433 (15)	0.0646 (18)	0.0013 (12)	0.0353 (15)	−0.0044 (14)
C9	0.0506 (16)	0.0481 (16)	0.0617 (17)	−0.0097 (12)	0.0319 (14)	−0.0089 (13)
C10	0.0452 (14)	0.0389 (14)	0.0516 (16)	−0.0021 (11)	0.0234 (12)	0.0027 (12)
C11	0.0405 (13)	0.0377 (14)	0.0455 (15)	0.0026 (10)	0.0177 (12)	−0.0018 (11)
C12	0.0565 (18)	0.0641 (19)	0.0531 (18)	0.0021 (14)	0.0179 (15)	0.0012 (14)
C13	0.0510 (18)	0.083 (2)	0.068 (2)	−0.0031 (16)	0.0060 (15)	−0.0138 (18)
C14	0.0438 (17)	0.067 (2)	0.091 (3)	−0.0118 (15)	0.0202 (18)	−0.0215 (19)
C15	0.0546 (17)	0.0498 (16)	0.080 (2)	−0.0072 (13)	0.0389 (17)	−0.0042 (15)
C16	0.0407 (14)	0.0403 (14)	0.0508 (15)	0.0024 (11)	0.0213 (12)	−0.0012 (12)
C17	0.078 (2)	0.0573 (18)	0.0635 (19)	0.0014 (15)	0.0398 (17)	0.0141 (15)
C18	0.108 (3)	0.069 (2)	0.0548 (19)	−0.005 (2)	0.0320 (19)	0.0149 (16)
N1	0.0442 (12)	0.0442 (13)	0.0620 (14)	−0.0060 (10)	0.0312 (11)	−0.0030 (11)
O1	0.0679 (14)	0.0947 (17)	0.0812 (16)	−0.0007 (12)	0.0382 (13)	0.0271 (14)
O2	0.0753 (14)	0.0670 (13)	0.0737 (14)	−0.0095 (11)	0.0348 (12)	0.0137 (11)
O3	0.0542 (11)	0.0581 (12)	0.0530 (11)	−0.0049 (9)	0.0213 (9)	0.0157 (9)
O4	0.0798 (17)	0.0854 (17)	0.0969 (19)	−0.0082 (13)	0.0190 (14)	0.0303 (14)

### Geometric parameters (Å, º)

**Table:**

C1—O1	1.205 (3)	C10—H10A	0.9700
C1—N1	1.393 (3)	C10—H10B	0.9700
C1—C2	1.483 (4)	C11—C12	1.373 (3)
C2—C7	1.372 (4)	C11—C16	1.390 (3)
C2—C3	1.378 (4)	C12—C13	1.378 (4)
C3—C4	1.382 (5)	C12—H12	0.9300
C3—H3	0.9300	C13—C14	1.356 (4)
C4—C5	1.366 (5)	C13—H13	0.9300
C4—H4	0.9300	C14—C15	1.379 (4)
C5—C6	1.382 (4)	C14—H14	0.9300
C5—H5	0.9300	C15—C16	1.386 (3)
C6—C7	1.373 (4)	C15—H15	0.9300
C6—H6	0.9300	C16—O3	1.369 (3)
C7—C8	1.482 (4)	C17—O3	1.424 (3)
C8—O2	1.208 (3)	C17—C18	1.488 (4)
C8—N1	1.379 (3)	C17—H17A	0.9700
C9—N1	1.455 (3)	C17—H17B	0.9700
C9—C10	1.515 (3)	C18—O4	1.419 (4)
C9—H9A	0.9700	C18—H18A	0.9700
C9—H9B	0.9700	C18—H18B	0.9700
C10—C11	1.498 (3)	O4—H4A	0.8200
			
O1—C1—N1	124.6 (3)	C12—C11—C16	117.5 (2)
O1—C1—C2	129.9 (3)	C12—C11—C10	121.9 (2)
N1—C1—C2	105.5 (2)	C16—C11—C10	120.6 (2)
C7—C2—C3	121.0 (3)	C11—C12—C13	122.3 (3)
C7—C2—C1	108.3 (2)	C11—C12—H12	118.9
C3—C2—C1	130.7 (3)	C13—C12—H12	118.9
C2—C3—C4	117.4 (3)	C14—C13—C12	119.2 (3)
C2—C3—H3	121.3	C14—C13—H13	120.4
C4—C3—H3	121.3	C12—C13—H13	120.4
C5—C4—C3	121.4 (3)	C13—C14—C15	120.9 (3)
C5—C4—H4	119.3	C13—C14—H14	119.6
C3—C4—H4	119.3	C15—C14—H14	119.6
C4—C5—C6	121.3 (3)	C14—C15—C16	119.2 (3)
C4—C5—H5	119.4	C14—C15—H15	120.4
C6—C5—H5	119.4	C16—C15—H15	120.4
C5—C6—C7	117.3 (3)	O3—C16—C15	124.6 (2)
C5—C6—H6	121.4	O3—C16—C11	114.5 (2)
C7—C6—H6	121.4	C15—C16—C11	120.9 (2)
C2—C7—C6	121.7 (3)	O3—C17—C18	105.9 (2)
C2—C7—C8	108.0 (2)	O3—C17—H17A	110.5
C6—C7—C8	130.3 (3)	C18—C17—H17A	110.5
O2—C8—N1	125.1 (2)	O3—C17—H17B	110.5
O2—C8—C7	128.8 (3)	C18—C17—H17B	110.5
N1—C8—C7	106.2 (2)	H17A—C17—H17B	108.7
N1—C9—C10	112.6 (2)	O4—C18—C17	112.0 (3)
N1—C9—H9A	109.1	O4—C18—H18A	109.2
C10—C9—H9A	109.1	C17—C18—H18A	109.2
N1—C9—H9B	109.1	O4—C18—H18B	109.2
C10—C9—H9B	109.1	C17—C18—H18B	109.2
H9A—C9—H9B	107.8	H18A—C18—H18B	107.9
C11—C10—C9	111.38 (19)	C8—N1—C1	112.0 (2)
C11—C10—H10A	109.4	C8—N1—C9	124.0 (2)
C9—C10—H10A	109.4	C1—N1—C9	124.1 (2)
C11—C10—H10B	109.4	C16—O3—C17	119.6 (2)
C9—C10—H10B	109.4	C18—O4—H4A	109.5
H10A—C10—H10B	108.0		
			
O1—C1—C2—C7	178.8 (3)	C11—C12—C13—C14	0.9 (4)
N1—C1—C2—C7	0.0 (3)	C12—C13—C14—C15	0.0 (5)
O1—C1—C2—C3	0.5 (5)	C13—C14—C15—C16	−0.8 (4)
N1—C1—C2—C3	−178.3 (3)	C14—C15—C16—O3	−179.5 (2)
C7—C2—C3—C4	0.5 (4)	C14—C15—C16—C11	0.8 (4)
C1—C2—C3—C4	178.6 (3)	C12—C11—C16—O3	−179.7 (2)
C2—C3—C4—C5	0.0 (4)	C10—C11—C16—O3	1.1 (3)
C3—C4—C5—C6	−0.1 (5)	C12—C11—C16—C15	0.0 (4)
C4—C5—C6—C7	−0.3 (4)	C10—C11—C16—C15	−179.2 (2)
C3—C2—C7—C6	−1.0 (4)	O3—C17—C18—O4	−65.5 (3)
C1—C2—C7—C6	−179.4 (2)	O2—C8—N1—C1	178.8 (2)
C3—C2—C7—C8	178.5 (2)	C7—C8—N1—C1	−0.1 (3)
C1—C2—C7—C8	0.0 (3)	O2—C8—N1—C9	−0.5 (4)
C5—C6—C7—C2	0.8 (4)	C7—C8—N1—C9	−179.3 (2)
C5—C6—C7—C8	−178.5 (2)	O1—C1—N1—C8	−178.9 (3)
C2—C7—C8—O2	−178.8 (3)	C2—C1—N1—C8	0.1 (3)
C6—C7—C8—O2	0.6 (5)	O1—C1—N1—C9	0.4 (4)
C2—C7—C8—N1	0.1 (3)	C2—C1—N1—C9	179.3 (2)
C6—C7—C8—N1	179.4 (3)	C10—C9—N1—C8	95.6 (3)
N1—C9—C10—C11	−171.9 (2)	C10—C9—N1—C1	−83.5 (3)
C9—C10—C11—C12	−96.2 (3)	C15—C16—O3—C17	−1.9 (4)
C9—C10—C11—C16	82.9 (3)	C11—C16—O3—C17	177.8 (2)
C16—C11—C12—C13	−0.9 (4)	C18—C17—O3—C16	179.7 (2)
C10—C11—C12—C13	178.3 (3)		

### Hydrogen-bond geometry (Å, º)

**Table:**

*D*—H···*A*	*D*—H	H···*A*	*D*···*A*	*D*—H···*A*
O4—H4*A*···O2	0.82	2.14	2.941 (3)	164
